# The Role of Propolis in Oxidative Stress and Lipid Metabolism: A Randomized Controlled Trial

**DOI:** 10.1155/2017/4272940

**Published:** 2017-04-30

**Authors:** Verónica Mujica, Roxana Orrego, Jorge Pérez, Paula Romero, Paz Ovalle, Jessica Zúñiga-Hernández, Miguel Arredondo, Elba Leiva

**Affiliations:** ^1^Escuela de Medicina, Universidad Católica del Maule, Talca, Chile; ^2^Departamento de Bioquímica Clínica e Inmunohematología, Facultad Ciencias de la Salud, Universidad de Talca, Talca, Chile; ^3^Programa de Investigación de Excelencia Interdisciplinario en Envejecimiento Saludable (PIEI-ES), Universidad de Talca, Talca, Chile; ^4^Escuela de Tecnología Médica, Facultad Ciencias de la Salud, Universidad de Talca, Talca, Chile; ^5^Research Department, Laboratorio Rotterdam Ltda, Talca, Chile; ^6^Laboratorio de Investigaciones Médicas, Escuela de Medicina, Universidad de Talca, Talca, Chile; ^7^Laboratorio de Micronutrientes, Instituto de Nutrición y Tecnología de los Alimentos (INTA), Universidad de Chile, Santiago, Chile

## Abstract

Although there is evidence of the benefits of propolis on human health, the vast majority of studies have been conducted using animal models. The present study includes the chemical characterization and clinical evaluation of the effects of the oral administration of propolis solution on the oxidative status and modulation of lipids in a human population in Talca, Chile. Chemical characterization of propolis, total phenol, flavonoids, and total antioxidant capacity were determined by ORAC. Identification of phenols and flavonoids in propolis was assessed by HPLC-DAD. A double-blind, placebo-controlled clinical trial was conducted. Subjects provided informed consent form and the Bioethics Committee of the Universidad de Talca approved protocol. Eligible subjects (*n* = 67) were randomized in two groups: propolis (*n* = 35) and placebo (*n* = 32). All subjects were evaluated at 0 (baseline), 45, and 90 days. In the propolis group, we observed that increases in HDL-c went from 53.9 ± 11.9 to 65.8 ± 16.7 mg/dL (*p* < 0.001) from baseline to 90 days. Compared to placebo subjects, consumption of propolis induced a net increase in GSH levels (*p* < 0.0001) and a decrease (*p* < 0.001) in TBARS levels for the propolis group. Our findings indicate potential benefits of propolis use in human health. The use of propolis appears to have positive effects on oxidative status and improvement of HDL-c, both of which contribute to a reduced risk of cardiovascular disease.

## 1. Introduction

Propolis is a sticky, resinous material that honeybees (*Apis mellifera* L.) collect from various plants and mix with wax and other secretions [1]. Numerous biological properties of propolis have been reported including cytotoxic, antimicrobial, antiviral, free radical scavenging, anti-inflammatory, local anesthetic, hepatoprotective, antitumor, and immune system stimulating [2, 3]. For these reasons, propolis is widely used in popular medicine and apitherapy, with extensive use in food and beverages to improve health and prevent diseases [3]. The medical application of propolis has led to increased interest in its chemical composition and potential clinical use in humans.

The chemical composition of propolis is complex and dependent upon the plant source [4]. Analysis of different propolis samples has identified at least 300 different compounds; biological activities are mainly attributed to the phenolic components such as flavonoids in all their forms (flavonols, flavones, flavonones, dihydroflavonols, and chalcones), terpenes, beta-steroids, aromatic aldehydes, and alcohols [5, 6]. The principal antioxidant mechanism of propolis polyphenols can be summarized in the significant ability of “scavenger” reactive oxygen species (ROS) and radical reactive nitrogen species (RNS) to decrease the xanthine oxidase reaction; chelate ion metals are involved in the process of free radical creation and disrupt the cascade of reactions, leading to the peroxidation of lipids and synergistic action with other antioxidants [7, 8].

It is well known that lipid peroxides are produced through a free radical chain process of autoxidation of lipids containing polyunsaturated fatty acids; their formation by ROS action has been implicated in the pathogenesis of various diseases [9, 10], such as atherosclerosis, myocardial infarction, diabetes mellitus type 2 (DM2), metabolic syndrome, and renal dysfunction [11]. The underlying mechanisms in disease development are different. In the case of atherosclerosis and cardiovascular complications, the primary risk factor is endothelial dysfunction, which is associated with LDL oxidation. For diabetes, beta-cell dysfunction and susceptibility to oxidative stress, which deplete insulin regulation, are fundamental. In renal disease, at the glomeruli and interstitial level, damage is associated with membrane oxidation and the favouring of albumin excretion and other relevant particles.

Previous studies have evaluated the role of propolis in carbohydrate metabolism. The following functions have been described: the epicatechin-mediated stimulation of insulin synthesis in pancreatic *β*-cells via increased cAMP [12], *β*-cell proliferation promoted by genistein, and, using in vivo analysis of epigallocatechin gallate, inhibition of glucose production by the liver [13]. Phenolics compounds can also influence glucose absorption in the gut by inhibiting *α*-amylase, *β*-glucosidase, and intestinal maltase [13, 14]. Furthermore, it has been reported that benzyl caffeate, isolated from propolis, inhibits the formation of lipid peroxides and very low doses of propolis ethanol extract exert an antilipid peroxidative action [15]. Also it is interesting to mention that not all the studies have shown successful result related to propolis administration [16], leaving open the opportunity to explore the mechanism and doses necessaries of propolis for human health.

It has also been described that propolis may prevent the rise of triglycerides (TG) as well as low and very low density lipoprotein cholesterol (LDL-c and VLDL-c). In an alloxan-induced rat model of type 2 diabetes mellitus [1], the regulation of lipid metabolism by propolis could be explained by its association with key proteins in lipogenesis and lipolysis, such as HMG-CoA reductase [17]. Recently, studies have indicated that the ethanolic extract of propolis and its subfractions are beneficial in increasing high-density lipoprotein (HDL) [17] and enhancing liver ATP-binding cassette transporters A1 and G1 (ABCA1 and ABCG1). This protein expression is associated with cholesterol efflux from peripheral tissue, suggesting that propolis may be involved in HDL particle formation and lead to an increase in plasma HDL [18, 19].

In light of the available knowledge about the potential benefits of propolis in human health, given its high phenolic profile it would be interesting to demonstrate the effect of propolis on the overproduction of free radicals (oxidative stress) in human beings. Taking into account the role of the oxidative stress in the genesis of several chronic human diseases (degenerative, cardiovascular, cancer, and any other pathologies), this research may contribute to their prevention.

Although there is evidence from traditional medicine about the benefits of propolis, there are few scientific works in human research that support it. Considering the previous information submitted and taking note of the differences observed between the chemical compositions among types of propolis from different geographical areas which depended on the surrounding flora, the present study reports on its chemical composition and provides a clinical evaluation of the effects of a propolis solution (administered orally) on the oxidative status and modulation of serum lipids in human subjects in Talca, Chile.

## 2. Materials and Methods

### 2.1. Propolis

Propolis (Beepolis®) is a 3% solution preparation in propylene glycol (PG), manufactured by a bee products company (Laboratorio Rotterdam Ltda) in the Maule Region, Chile (Health Authorization n° 639-18/08/2009, granted by Ministry of Health Regional Office, Maule Region).

### 2.2. Chemical Analysis of Propolis

#### 2.2.1. Total Phenolic Content (TPC)

The TPC of the propolis solution (propolis dissolved in propylene glycol) was determined according to the Folin-Ciocalteu method [20]. Briefly, 20 *µ*L of sample or standard (gallic acid, GAE) was mixed with 1.58 mL of distilled water and 100 *µ*L of Folin-Ciocalteu reagent. The reaction mixture was preincubated for 8 min and then 300 *µ*L of sodium carbonate 20% was added. Finally, each tube was incubated for 2 h at room temperature and the absorbance was obtained in a spectrophotometer (Thermo Spectronic Genesys 10 UV) at a wavelength of 765 nm. The TPC was expressed as GAE in grams per 1000 ml of sample.

#### 2.2.2. Total Flavonoid Content (TFC)

The TFC propolis was determined spectrophotometrically using the method reported by Zhishen et al. 1999 [21], based on the formation of a flavonoid-aluminum complex. Briefly, 0.5 mL of propolis solution (3% propolis dissolved in propylene glycol) or standard (quercetin) was mixed with 2 mL of distilled water and 0.15 mL of sodium nitrate (NaNO_3_, 5%). After 6 min of incubation, 0.15 mL of aluminum chloride (AlCl_3_, 10%) was added and allowed to incubate for another 6 min, after which 2.0 mL of sodium hydroxide (NaOH, 4%) was added to the mixture. Water was added to achieve a final volume of 5 mL, and the solution was incubated for another 15 min. Absorbance was obtained in a spectrophotometer (Thermo Spectronic Genesys 10 UV) at a wavelength of 510 nm. The results were reported as quercetin equivalents (QE) in milligrams per 1000 ml of sample.

#### 2.2.3. Antioxidant Capacity by Oxygen Radical Absorption Capacity (ORAC) Assay

The antioxidant capacity method was adapted from Dávalos et al. [22]. Briefly, different dilutions of propolis solution (3% propolis dissolved in propylene glycol) or Trolox (standard) were placed in a microplate containing 21 *μ*M fluorescein in 75 mM phosphate buffer, pH 7.4. The mixture was preincubated for 20 min at 37°C, and then 19 mM of 2,2′-azobis(2-aminopropane) (ABAP) was added. Fluorescence intensity (*λ*exc = 485 nm, *λ*em = 512 nm) was registered in a Varioskan Flash microplate reader (Thermo Electron Corp.). The Trolox equivalent concentration for propolis solution was obtained from the calibration curve (the standard curve was obtained by plotting the net area under the curve (AUC) of different Trolox concentrations). ORAC values were calculated using the difference between the area under the fluorescein decay curve and the blank (net AUC). Regression equation between net AUC and antioxidant concentration was calculated for the sample. ORAC values were expressed as *μ*mol of Trolox equivalents per gram of propolis solution.

#### 2.2.4. Compound Identification by HPLC-DAD

Chromatography was assayed according to Pellati et al. [23]. Determination was performed using an Agilent Technologies (Waldbronn, Germany) modular model 1100 system with a diode array detector (DAD). The chromatograms were recorded using Agilent ChemStation for LC and LC-MS systems. The analyses were carried out using Ascentis C18 column (250 mm × 4.6 mm ID, 5 *μ*m, Supelco, Bellefonte, PA, USA). The mobile phase was composed of (A) 0.1% formic acid in H_2_O and (B) ACN. The postrunning time was 5 min. The flow rate was 1.2 mL/min. The column temperature was set at 30°C. The sample injection volume was 5 *μ*L. The DAD acquisitions were performed in the range 190–450 nm. The sample preparation for HPLC analysis consisted of 500 *μ*L of propolis and was diluted with MeOH, filtered through a 0.45 *μ*m PTFE (polytetrafluoroethylene) filter into an HPLC vial, and injected into the HPLC system. All sample preparations were carried out in duplicate. The standard solution of each compound (aldehyde benzoic acid, caffeic acid, *p*-coumaric acid, ferulic acid, quercetin, pinobanksin, cinnamic acid, apigenin, veratric acid, and vanillin, among others) was prepared as pure compound (2–6 mg) in MeOH. The external standard calibration curve was generated using five data points. Five *μ*l aliquots (in triplicate) of each standard solution were used for HPLC analysis.

### 2.3. Subjects

**Figure 1:**
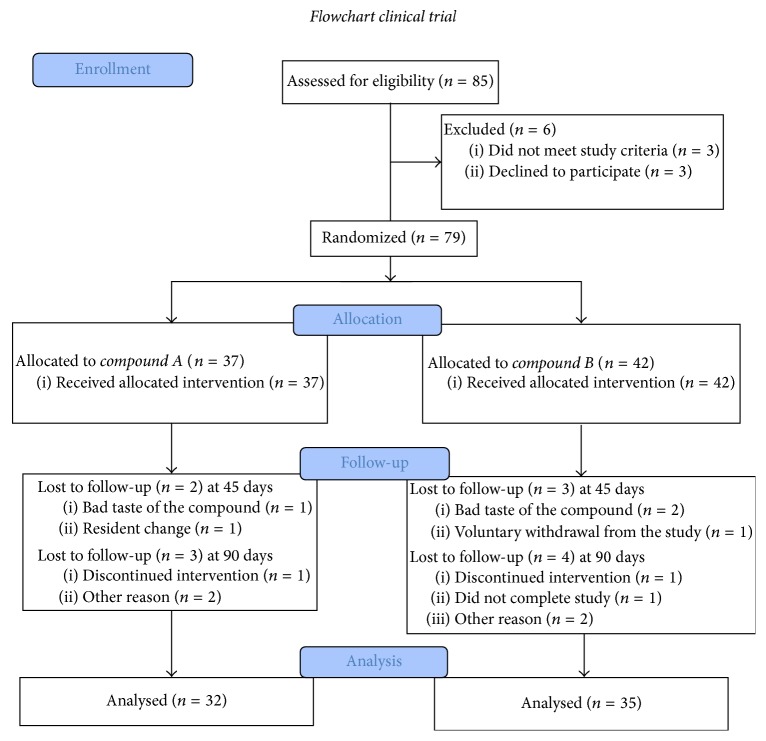
Eligibility, randomization, and patient follow-up.* Compound A* is placebo and* compound B* is propolis.

This clinical trial was a randomized, double-blind, and placebo-controlled. Subjects were invited to participate in the study via an institutional email (Universidad de Talca, Talca, Maule, Chile) and 85 subjects were interested in this clinical trial. The subject flowchart is shown in Figure 1. The first subjects were enrolled between March 19 and May 26, 2014. Follow-up was from May 29 to September 11, 2014.

*The inclusion criteria were as follows*: (i) an age range of 18–69 years; (ii) having at least one of the following altered parameters: fasting glycemia, lipids profile, blood pressure, or diabetes mellitus, cardiovascular disease, and/or overweight. The exclusion criteria were as follows: (i) history of significant alcohol consumption; (ii) reported acute or chronic pathological conditions (liver and/or renal failure, uncontrolled diabetes mellitus, or immunodeficiency and immunological disorders, among others); (iii) being unlikely to cooperate with the study regime. During the study, 8 subjects withdrew voluntarily and 3 additional subjects were excluded for having insulin above the normal range (>100 *µ*U/mL).

### 2.4. Ethics Statement

The study was performed in compliance with ethical principles and good clinical practice. All subjects provided a written informed consent prior to participation in the study, approved by the Bioethics Committee of the Universidad de Talca (Page Number 2013-064, November 2013).

### 2.5. Treatment Groups

Eligible subjects were randomized in two groups (A or B) using a Microsoft Excel spreadsheet. Only Rotterdam Laboratory knew the meaning of the codes. They sent two sets of bottles with identical physical characteristics (shape, size, and color), marked only with a single letter code (A or B). One group consumed propolis (*n* = 35) and the other (*n* = 32) a placebo with similar flavours (mixture of peppermint, fernet, and synthetic). The propolis and placebo were administered orally twice daily in the same dose and formulation (15 drops each time) for 90 days. At the beginning and during the course of the study the subjects were evaluated for allergic reactions, epigastric discomfort, and any other adverse reaction at 0 (baseline), 45, and 90 days. They were also monitored by phone, focus group, and an in-person interview. All assays were performed according to international standards used in clinical laboratories, which include a calibration curve for each analyte (*r* = 0,999) and internal quality controls protocols. Blood pressure was taken twice on every measure day (0, 45, and 90), after sitting 5 minutes, and then again after 10 minutes, and the average was used in the analysis. All analyses were conducted comparing baseline to day 90. At the end of the study, the results were unblinded.

### 2.6. Anthropometric and Blood Pressure Measurements

Height was measured to the nearest 0.5 cm and weight to the nearest 0.1 kg using a mechanical column scale with eye-level beam (Seca 220®, Ca, USA). BMI was classified based on age and sex norms for underweight, normal, overweight, or obese cases (<18.5, 18.5 to 24.9, 25 to 29.9, and >30, resp.). Waist circumference (cm) was measured at the midpoint between the lower ribs and the iliac crest. Hip circumference was measured horizontal at the largest circumference of the hip. Blood pressure (mmHg) was measured in an Omron® digital sphygmomanometer (Osaka, Japan).

### 2.7. Fasting Blood Samples

These were used to measure the levels of glucose by colorimetric enzymatic hexokinase reagent kit (Glucose-Custom Biotech), insulin (by electrochemiluminescence immunoassay, Insulin ECLIA), and glycosylated haemoglobin (HbA1c, by a turbidimetric inhibition immunoassay, Tina-quant haemoglobin A1c Gen.2®). The lipid profile was determined: (i) total cholesterol by using the CHOD-PAP enzymatic colorimetric test (Cholesterol Gen.2®); (ii) triglycerides (TG) by GPO-PAP enzymatic colorimetric test (Triglycerides GPO/PAP®); (iii) HDL-c by enzymatic colorimetric test (HDL-Cholesterol plus 3rd generation®). The liver enzymatic profile was determined: (i) *γ*-glutamyltransferase by enzymatic colorimetric test (*γ*-Glutamyltransferase ver.2®); (ii) alanine aminotransferase (ALT) and aspartate aminotransferase (AST) by quantitative determination of the catalytic activity by colorimetric enzymatic assay (ALT or AST acc. to IFCC without pyridoxal phosphate activation®); and C-reactive protein (CRP), by highly sensitive turbidimetric immunoassay (Cardiac C-Reactive Protein [Latex] High Sensitive®). All analyses were measured in a Cobas c311 autoanalyser Roche (Zurich, Switzerland). LDL-c (mg/dL) was calculated according to Friedewald's protocol [24]: [total cholesterol (mg/dL) − (HDL-c (mg/dL) + TG (mg/dL)/5)]. The homeostatic model assessment of insulin resistance (HOMA-IR) was calculated according to Blümel et al. [25]: HOMA-IR = [(glycemic mg/dl) × (insulinemic *µ*U/mL)/405].

*Clinical Parameters of Oxidation*. To determine oxidative damage, TBARS were measured during an acid-heated reaction as previously described [26]. Serum (0.3 mL) was mixed with 180 *µ*L of trichloroacetic acid 50% and 600 *µ*L of thiobarbituric acid 0.67% and then heated in a boiling water bath (90°C) for 30 min. Malondialdehyde (MDA) equivalents were determined by calibration curve (1–10 nmol MDA/mL of sample). TBARS were determined spectrophotometrically (Multiskan Go, Thermo Scientific, Massachusetts, USA) at 532 nm and expressed as nmol MDA/mL of sample. Total levels of GSH were determined according to Beutler et al. [27]; 40 *µ*L of total blood plus 760 *µ*L of water was added to 1200 *µ*L of protein precipitant reactive (1.67 g metaphosphoric acid, 0.2 g EDTA, and 30 g NaCl in a final volume of 100 mL of distilled water); then the mixture was centrifuged at 3500 rpm for 10 min. The supernatant was collected and mixed with 125 *µ*L of DTNB 5,5′-dithiobis(2-nitrobenzoic acid) in 0.4% buffer sodium phosphate 0.1 M, pH 7.5. After 5 minutes the samples were measured spectrophotometrically at 412 nm.

### 2.8. Statistical Analysis

All the data were evaluated using the by Shapiro-Wilk test for normality of the variable. Values correspond to the mean ± standard deviation (SD). The statistical analysis included intragroup *t*-test analysis and one-way ANOVA followed by Tukey's posttest. A *p* value of <0.05 was considered statistically significant. The data were evaluated with GraphPad Prism 6® software (La Jolla, CA, USA).

## 3. Results

### 3.1. Total Phenolic and Flavonoid Compounds, Antioxidant Capacity, and Chemical Identification of Propolis Compounds by HPLC-DAD

**Figure 2:**
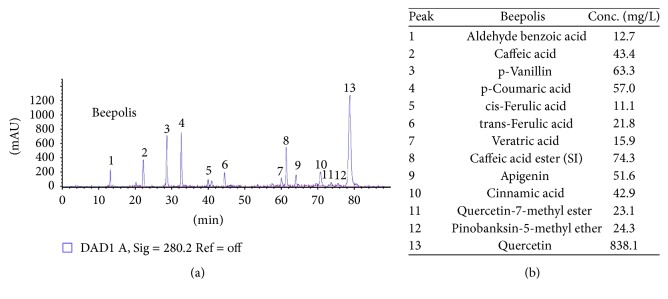
*Chemical characterization of propolis*. Chromatogram obtained by HPLC-DAD analysis of a propolis sample at 280 nm (a). Peak information of the chromatogram (b).

Table 1 shows the TPC, TFC (flavonols plus OH-flavonols), and ORAC values for the propolis assayed. The HPLC-DAD analysis of the commercial sample of propolis measured at 280 nm denoted a complex composition, as shown in Figure 2(a). The chromatogram of each characterized compound is presented by a number over each chromatographic peak. The corresponding peak identification is described in Figure 2(b). Thirteen different main compounds were identified and ordered from highest to lowest concentration: quercetin > caffeic acid ester >* p*-vanillin >* p*-coumaric acid > apigenin > caffeic acid > cinnamic acid > pinobanksin-5-methyl-ether > quercetin-7-methyl-ester >* trans*-ferulic acid > vetranic acid > aldehyde benzoic acid > cis-ferulic acid.

### 3.2. Description of General Characteristics

A total of 85 subjects were eligible for this study, 79 of whom provided informed consent and were randomized.  Table 2 summarizes the demographics characteristics of the study population. The placebo group was comprised of 7 men and 25 women with an average age of 44.5 ± 13.7 years, a weight of 74.5 ± 14.4 kg, and a height of 162 ± 8 cm. Average BMI was 28.2 kg/m^2^. The propolis group was made up of 9 men and 26 women with an average age of 48 ± 12.1 years, a weight of 69.6 ± 12.5, a height of 162 ± 8 cm, and a BMI of 27.9 ± 4.8. Both groups were, on average, overweight. In terms of weight, BMI, and waist circumference there were no significant differences between the groups at baseline or 90 days. All the anthropometric variables analysed are shown in Table 3. In the propolis group systolic and diastolic blood pressure saw a significant reduction: SBP from 126.1 ± 9.5 to 121.9 ± 9.3 mmHg and DBP from 79.4 ± 10.2 to 76.2 ± 6.9 (*t* test; *p* < 0.018).

### 3.3. Carbohydrate Metabolism and Liver Profile in the Study Group

Fasting glycaemia, HbA1c, and insulin were measured at day 0 and 90 (Table 4), with no significant changes within the groups: blood sugar levels and insulin were stable over time and within normal limits. We calculated the HOMA index and considered the HOMA-IR > 2.5 as a cutoff to determine insulin resistance; HOMA values decreased in the propolis group, from 2.54 ± 1.91 (baseline) to 2.43 ± 1.28 (day 90), but differences were not statistically significant. Analysis of liver enzymatic activity (GGT, GOT, and GPT) did not show any variations within or between groups (Table 5). No signs of allergy or other adverse reactions to propolis consumption were observed among the study participants.

### 3.4. Lipids Profile in Study Group

**Figure 3:**
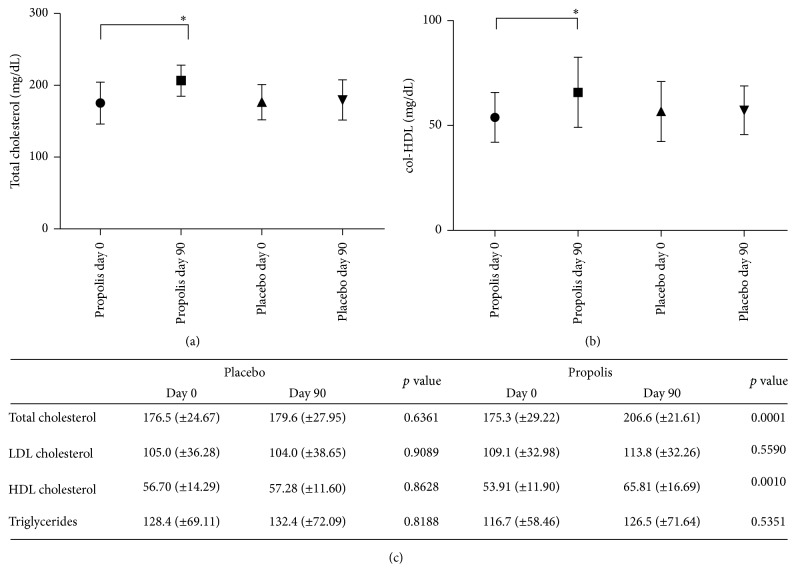
*Lipids profile in study group*. Plasma total cholesterol graph (a), HDL-c graph (b), and table of total cholesterol, LDL-c, HDL-c, and triglycerides (c), all determined by enzymatic methods. Values correspond to mean ± SD for 32 placebo subjects and 35 propolis subjects (one-way ANOVA, Tukey's posttest ^*∗*^*p* < 0.05).

The effects of propolis on blood lipids in human subject are given in Figure 3. The propolis group had a 17% increase in total cholesterol (Figure 3(a)) at day 90 from 175.3 ± 29.2 to 206.6 ± 21.6 mg/dL (one-way ANOVA, Tukey posttest: *p* < 0.0001) and a 22% increase in HDL-c (Figure 3(b)) from 53.9 ± 11.9 to 65.8 ± 16.7 mg/dL (one-way ANOVA, Tukey posttest: *p* < 0.001), compared with day 0 of propolis administration and with the placebo subjects. There were no statistically significant differences in LDL-c (intragroup *t* Test: *p* > 0.559) and TG (Intragroup *t* Test: *p* > 0.535) in the propolis group; additionally the placebo group did not show any variation in the lipid parameters measured (see Figure 3(c)).

### 3.5. Oxidative Parameters in Study Group

**Figure 4:**
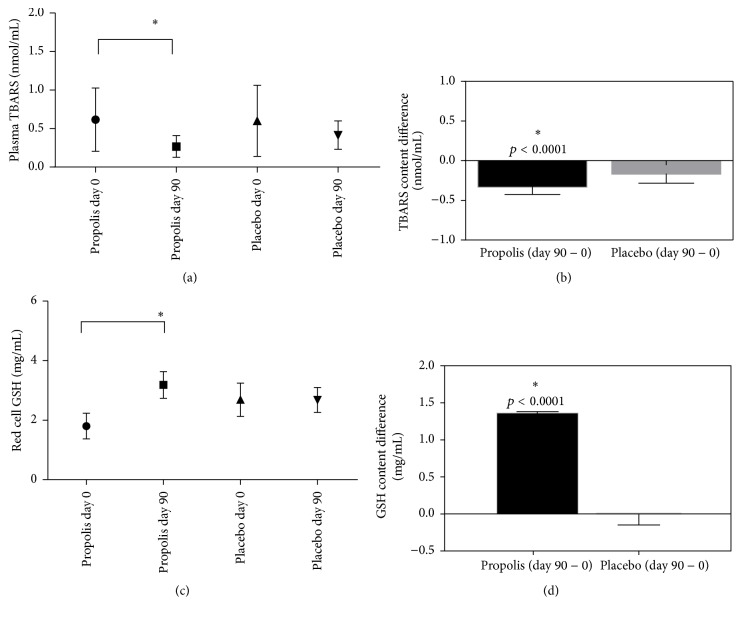
*Oxidative parameters in study group*. Oxidative stress was assayed by TBARS (a), net changes (day 90 − 0) in TBARS (b), reduced glutathione GSH (c), and net changes (day 90 − 0) in GSH (d). Values correspond to mean ± SD for 32 placebo subjects and 35 propolis subjects. Significance was evaluated by one-way ANOVA and Tukey's posttest ((a) and (c) ^*∗*^*p* < 0.05) or *t* Test ((b) and (d) ^*∗*^*p* < 0.05).

In the propolis group, TBARS decreased by 67% (one-way ANOVA, Tukey's posttest: *p* < 0.0001) and GSH levels increased by 175% (one-way ANOVA, Tukey's posttest: *p* > 0.0002), with both changes being observed at day 90 compared with day 0 of intake (Figures 4(a) and 4(c), resp.). Among subjects who received the placebo, plasma levels of TBARS and GSH were comparable throughout the study (0 to 90 days), with nonstatistically significant changes. TBARS had a net decrease in subjects in the propolis group, which was higher than that observed in the placebo group (*t* Test: *p* < 0.0001; see Figure 4(b)). There was a net increase of GSH levels (*t* Test: *p* < 0.0001), in propolis versus placebo subjects (Figure 4(d)).

## 4. Discussion

In the last years the interest in functional foods from natural origin for quality of life improvement and disease prevention has increased. Some of these functional foods derived from the hives industry (e.g., honey and propolis). Traditional knowledge has shown benefits when consuming these products, and there is an abundance of scientific work characterizing propolis from different parts of the world and its effects in vitro on cells or rats. There are very few clinical studies, however, that demonstrate conclusively the health effects in human beings. Propolis has traditionally been used to treat infections, but scientific evidence of its value as an antioxidant and/or in the management of chronic diseases such as diabetes, atherosclerosis, and cancer is insufficient. Propolis has a large number of bioactive compounds: a variety of polyphenols and flavonoids, related to the flora surrounding the hives [4]. Nina et al. (2015) described a large variation in in vitro antimicrobial effects, antioxidant activity, and composition in four different geographic areas of propolis from the Maule Region of Chile. Researchers found that propolis sample from the central valley was more effective as an antibacterial than those from the coastal range or Andean slopes [28]. Bankova et al. (2014) analysed the chemical composition and antiviral activity of commercial propolis Extract ACF® (PPE) (ethanolic extract at a concentration of 3%). They showed that PPE had a high antiviral activity against herpes simplex virus type 1 and type 2 which may partly be due to interference in the viral adsorption to the cells [29]. Miyazaki et al. (2015) worked with Brazilian ethanol extracts of propolis, evaluating their action in oxidative stress in both in vivo and in vitro studies, related to the cognitive dysfunction associated with hyperhomocysteinemia. This study found that propolis improved cognitive function, decreasing the accumulation of proteins in the brain, mediated by an increase in homocysteine [30]. On the other hand, Chilean studies with propolis from the Araucanía region showed a modulation of the angiogenesis in both in vivo and in vitro models. Cuevas et al. (2015) showed that ethanolic extracts of Chilean propolis, specifically pinocembrin, one of its main constituents, were able to modulate in vitro angiogenesis, in part by modulating HIF1*α* stabilization and ERK1/2 phosphorylation, two important factors involved in this process. Other studies have evaluated in vitro the inhibitory activity of 22 propolis extracts from different Chilean regions on 10 strains of* Helicobacter pylori* isolated from gastric mucosa in vitro. The results show that Chilean propolis has an effective anti-*Helicobacter pylori* activity [31, 32].

The principal aim of this trial was to evaluate the most relevant effects associated with propolis, like oxidative status, lipid content, and carbohydrate level normalization in a placebo-controlled human study. First, we evaluated the total phenolic and flavonoid content. Compared to tropical zone propolis, the ORAC antioxidant capacity was higher [33]. This may be related to the particular characteristics of bioactive compounds, as the variety of polyphenols and flavonoids related to the flora surrounding the hives as a function of botanical and geographical origin [4]. Propolis types found in tropical areas contain a wide variety of phenolic compounds, such as* p*-coumaric acid, flavan-3-ol-flavonols, chalcones, isoflavonoids, pterocarpans, and triterpenoids, among others [33, 34]. In addition, Brazilian propolis has a high content of formononetin, isoliquiritigenin, pinocembrin, biochanin A, and quercetin [33, 35], a phenolic pattern that has many differences with the Chilean propolis assayed in this study, explaining the phenolic and flavonoids differences found among propolis.

The antioxidant capacity was evaluated by ORAC and the flavonoid and phenolic content are directly related with that observed in this clinical study, showing decreases in TBARS and GSH enhancement. Propolis has the capacity to reduce ROS, which could be related to two different mechanisms. According to the literature, the first is the capacity of caffeic acid phenethyl ester (CAPE) to activate the transcription factor NrF2 [36]. NrF2 is a regulatory protein associated with antioxidant protection and with the enhancement of antioxidant enzymes like heme oxygenase-1, phase II detoxification enzymes, and enzymes involved in GSH metabolism [36, 37]. Thus, through the phenolic compound propolis could activate NrF2 and improve cellular antioxidant capacity. The second mechanism could be triggered by the ability of the phenolic and flavonoid compounds like quercetin, CAPE,* p*-vanillin,* p*-coumaric acid, apigenin, and cinnamic acid, all of which are present in Chilean propolis, to neutralize oxidative species [38]. CAPE not only has been shown to inhibit activation of the nuclear transcription factor- (NF-) *κ*B signaling pathway [39], but also has strong ROS scavenging ability and activates NrF2 [40], thereby increasing an antioxidant stress response, which could in part explain the antioxidant effects observed in our study related to the increase in GSH and decrease of TBARS.

Other propolis components have been studied. Pinocembrin (5,7-dihydroxyflavanone), abundant flavonoid in propolis, has been shown to have antioxidant activity related to the nuclear translocation of NrF2, activation of the NrF2/ARE pathway, and induction of HO-1 and *ϒ*-GCS expression, which is related to the biosynthetic pathways of GSH formation [41]. The work of Ishige et al. (2001) [42] shows that flavonoids can deplete intracellular ROS indirectly by increasing intracellular GSH. Additionally, propolis can enhance glutamate-cysteine ligase, a rate-limiting enzyme in GSH synthesis [43], and it is therefore associated with strong free radical scavenging activities and improvement of the endogenous antioxidant defense system observed by propolis consumption.

In relation to the effects observed in HDL-c, it is important to highlight that this lipoparticle helps protect against cardiovascular disease [44–46], avoiding LDL oxidation or neutralizing the atherogenic effects of the oxidized-LDL in artery walls [5]. Currently, there are no approved drugs in therapeutics protocols to improve HDL-c levels or such drugs are less controversial. Recent studies have indicated that the ethanolic extract of propolis and its subfractions are beneficial for increasing plasma HDL-c while reducing LDL-c in a model of hypercholesterolemic rabbits [18]. According to the blood plasma analysis Brazilian propolis reduced total cholesterol and elevated HDL-c in LDLr−/− in mice with an initial atherosclerotic lesion [47]. Propolis enhances liver ATP-binding cassette transporters A1 and G1 (ABCA1 and ABCG1) protein expression, which is associated with cholesterol efflux from peripheral tissue. This suggests that propolis may be involved in HDL particle formation and may lead to an increase in HDL [18, 19]. Together with an increase of ABCA1 cassette, Brazilian red propolis upregulated ApoA-1-mediated cholesterol efflux by macrophages, an action related to ABCA1 via induction of PPAR*ϒ*/LXR, an important transcription factor related to lipid metabolism. The relationship of propolis with the lipid metabolism is a good indicator of its potential as a cardiovascular protector [48].

In relation to blood pressure, our findings showed a significant decrease in DBP in both the propolis and placebo groups, and we therefore estimate that these findings are likely only placebo effect. SBP decreased significantly but only in propolis group. Nevertheless, some evidence reported in the literature has described dietary antioxidants as possibly having beneficial effects on hypertension, although this has not been proven with antioxidant supplementation [49]. On the other hand, Teles et al. (2015) [50] demonstrated in an animal model that the antioxidant and anti-inflammatory effects of propolis were able to attenuate hypertension and structural renal damage in Wistar rats models. The reduction in PAS in the study group that took propolis was modest and therefore of uncertain clinical significance, but under the above background it would be interesting to reevaluate future effect on blood pressure in a study designed for hypertension patients. We found no differences in glucose, HbA_1-c_, or insulin levels. This may be due to the short intervention period, which underscores the importance of future studies over a more prolonged period to detect clinically relevant changes related to propolis consumption. Previously, Babatunde et al. (2015) [51] noted a significant decrease in blood glucose level in alloxan-induced hyperglycemia Wistar rats when given Nigerian propolis, suggesting that long-term administration/intake of this extract may have hypoglycemic effect. Blood glucose reduction may relate to the bioactive compounds of propolis on *β*-cells, which could enhance the production of insulin or enhance cellular sensitivity response to insulin.

## 5. Conclusion 

Data reported here support the role of propolis in diverse chronic disease, through different mechanisms such as the increase in HDL-c, and the antioxidant effect due to enhanced GSH and decreased TBARS levels, both markers of oxidative stress in humans. Therefore, our findings provide highlighted scientific information in using propolis as an antioxidant agent.

According to the results, the use of propolis may improve the prognosis of several chronic diseases and potentially contribute to decreasing the risk of cardiovascular disease.

## Figures and Tables

**Table 1:** Total phenolic and flavonoids content and antioxidant capacity of propolis.

	Beepolis
TPC (g GAE/L)	22.82
ORAC (*µ*mol equiv. Trolox/g)	42.73
TFC (mg quercetin/L)	937.1

**Table 2:** Demographic characteristics.

Demographics	Total (*n* = 67)	Placebo (*n* = 32)	Propolis (*n* = 35)
Age (mean ± SD)	46.4 (±12.9)	44.5 (±13.7)	48.0 (±12.1)
Gender			
Female	51 (76%)	25 (78%)	26 (74%)
Male	16 (24%)	7 (22%)	9 (26%)

**Table 3:** Effects of propolis over anthropometric and blood pressure measures.

	Placebo		*p* value	Propolis		*p* value
	Day 0	Day 90		Day 0	Day 90	
Weight (kg)	74.5 (±14.4)	74.8 (±14.4)	0.174	69.6 (±12.5)	68.7 (±11.6)	0.488
BMI (kg/m^2^)	28.2 (±4.7)	28.4 (±4.7)	0.182	27.9 (±4.8)	27.9 (±4.7)	0.409
Waist circumference (cm)	89.8 (±8.9)	91.1 (±9.2)	0.152	87.6 (±9.8)	87.9 (±9.2)	0.353
SBP (mmHg)	122.2 (±11.1)	121.6 (±11.6)	0.401	126.1 (±9.5)	121.9 (±9.3)	0.018^*∗*^
DBP (mmHg)	77.5 (±8.2)	74.6 (±9.3)	0.006^*∗*^	79.4 (±10.2)	76.2 (±6.9)	0.036^*∗*^

Values represent the mean ± SD for 32 placebo and 35 propolis subjects. Significant differences between the groups are indicated by a single asterisk (intragroup *t* test: *p* < 0.05). BMI: body mass index; SBP: systolic blood pressure; DBP: diastolic blood pressure.

**Table 4:** Carbohydrate metabolism in study group.

	Placebo		*p* value	Propolis		*p* value
	Day 0	Day 90		Day 0	Day 90	
Glycemia (mg/dL)	92.3 (±8.1)	95.4 (±7.6)	0.174	94.8 (±11.8)	97.9 (±10.8)	0.488
HbA1c (%)	5.49 (±0.35)	5.42 (±0.39)	0.487	5.50 (±0.35)	5.46 (±0.33)	0.194
HOMA	2.54 (±1.21)	2.58 (±1.12)	0.551	2.54 (±1.91)	2.43 (±1.28)	0.512
hs-CRP (mg/L)	2.05 (±1.30)	2.17 (±1.33)	0.465	2.02 (±1.09)	1.81 (±1.11)	0.676

Values represent mean (±SD) for 32 placebo and 35 propolis subjects. Nonsignificant differences between the groups were observed (intragroup *t* Test: *p* < 0.05); HOMA: homeostatic model assessment.

**Table 5:** Liver enzyme profile in the study group.

	Placebo		*p* value	Propolis		*p* value
	Day 0	Day 90		Day 0	Day 90	
GGT (U/L)	21.3 (±5.7)	18.9 (±7.0)	0.178	20.3 (±9.1)	18.4 (±6.7)	0.372
GOT (U/L)	19.0 (±2.8)	19.7 (±3.0)	0.383	20.6 (±2.9)	20.1 (±3.5)	0.554
GPT (U/L)	23.9 (±8.8)	22.0 (±6.3)	0.406	22.9 (±7.8)	20.3 (±6.7)	0.167

Values represent mean ± SD for 32 placebo subjects and 35 propolis subjects. Nonsignificant differences were found between the groups (intragroup *t* Test: *p* > 0.05). GGT: gamma glutamyl transferase; GOT: glutamic oxaloacetic transaminase; GPT: glutamate-pyruvate transaminase.
